# Improvement of the management of mental well-being and empathy in Chinese medical students: a randomized controlled study

**DOI:** 10.1186/s12909-021-02813-6

**Published:** 2021-07-10

**Authors:** Rong Rong, Wei Chen, Zihao Dai, Jingli Gu, Weiying Chen, Yanbin Zhou, Ming Kuang, Haipeng Xiao

**Affiliations:** 1 Clinical Trials Unit, the First Affiliated Hospital, Sun Yat-sen University, 510080 Guangzhou, China; 2 Department of Nephrology, the First Affiliated Hospital, Sun Yat-sen University, 510080 Guangzhou, China; 3 Department of Liver Surgery, the First Affiliated Hospital, Sun Yat-sen University, 510080 Guangzhou, China; 4 Department of Hematology, the First Affiliated Hospital, Sun Yat-sen University, 510080 Guangzhou, China; 5 Department of Diagnostics, the First Affiliated Hospital, Sun Yat-sen University, 510080 Guangzhou, China; 6 Department of Pulmonary and Critical Care Medicine, the First Affiliated Hospital, Sun Yat-sen University, 510080 Guangzhou, China; 7 Department of Medical Education, Department of Liver Surgery, College of Basic Surgery, Zhongshan School of Medicine, the First Affiliated Hospital, Sun Yat-sen University, No.58, Zhongshan Road 2, 510080 Guangzhou, People’s Republic of China; 8 Department of Medical Education, Department of Endocrinology, Zhongshan School of Medicine, the First Affiliated Hospital, Sun Yat-sen University, No.58, Zhongshan Road 2, 510080 Guangzhou, People’s Republic of China

**Keywords:** Empathy, Depression, Medical education, Medical students, Mental health

## Abstract

**Background:**

Among Chinese medical students, there is a high prevalence of mental health-related issues and low empathy. Effective strategies to improve this situation are lacking. This study aims to investigate the efficacy of the intervention courses designed to enhance the mental health and empathy of senior Chinese medical students.

**Methods:**

A total of 146 3rd - and 4th -year medical students were randomized to an intervention group (*n* = 74) and a control group (*n* = 72). A pilot study including 5 pre-clinical students and 5 interns was first carried out to determine the themes and content of the intervention courses. The designed courses were delivered in the intervention group once a month three times, while the control group had no specific intervention. Five self-assessment questionnaires, including the General Self-Efficacy (GSE) scale, Medical Outcomes Study Short Form 8 (SF-8), Patient Health Questionnaire-9 (PHQ-9), Maslach Burnout Inventory (MBI), and Jefferson Scale of Empathy-Health Care Provider Student version (JSE-HPS), were completed by the students before and one month after the courses to evaluate their levels of self-efficacy (SE), quality of life (QoL), depression, burnout, and empathy, respectively. Qualitative data were collected via e-mail two years after the intervention.

**Results:**

Compared to the control group, the intervention group showed significantly higher scores for empathy (111.0 [IQR 102.0, 118.0] vs. 106.0 [IQR 93.0, 111.5]; *P* = .01) and QoL (32.0 [IQR 28.0, 35.0] vs. 29.5 [IQR 26.0, 34.0]; *P* = .04). The rate of depression was significantly lower in the intervention group than in the control group (13.5 % vs. 29.2 %; chi-square test, *P* = .02). However, no significant differences in self-efficacy (25.6 ± 4.8 vs. 24.3 ± 6.3; *P* = .16) or burnout (27.0 % vs. 34.7 %; Chi-square test, *P* = .31) were observed between the two groups.

**Conclusions:**

The intervention courses had a positive impact on mental well-being and empathy in senior Chinese medical students, which might help provide novel information for their incorporation into the medical school curriculum.

**Trial registration:**

*ClinicalTrials.gov Identifier*: NCT02645643; *Date of registration*: 05/01/2016.

**Supplementary Information:**

The online version contains supplementary material available at 10.1186/s12909-021-02813-6.

## Background

Many stress factors, including long study hours, frequent examinations and deteriorating patient-physician communication, make medical students vulnerable to mental health problems [1–4]. Accumulating literature has shown that a significant proportion of medical students have different degrees of mental health problems [5, 6]. In addition, the prevalence of depression has been found to be significantly higher among medical students (22–36 %) than among precollege students and the general population (2–16 %) [7–10]. It has also been well documented that medical students are more susceptible to burnout associated with a noticeable lack of self-efficacy (SE), which affects their academic performance, clinical empathy and overall quality of life (QoL) [11]. In China, these mental health-related issues have been further exacerbated by the deteriorating patient-doctor relationship, as reflected by the intensive violent incidents perpetrated by the patients in the hospitals [12, 13]. Frequent medico-legal disputes have generated a heavy psychological burden and stress for medical students in China [14, 15] and have negatively impacted students’ passion and confidence in pursuing their future medical careers [13, 16]. The lack of communication skills due to a low level of empathy is an important factor contributing to a tense patient-physician relation in China [17, 18]. In addition, reduced empathy further compromises students’ clinical ability and performance [14, 15].

Although many previous reports have raised extensive concerns regarding the mental health of medical students, effective intervention strategies are lacking [16]. In recent years, several randomized controlled studies have investigated the ability of interventions using mindfulness-based stress reduction (MBSR) to reduce mental distress and burnout and improve coping skills [19, 20]. In addition, various interventions, including yoga, psychological counselling and Williams LifeSkills training, have also been reported to have positive effects on the improvement of stress, depression and quality of life [2, 21–23]. While these studies provide important data for our understanding of the modulation of mental health, there are some limitations in the work. Previous trials often focused on the short-term effects; nevertheless, the long-term effects of the designed interventions were unclear. More importantly, most of the interventions were designed without detailed research on the mental difficulties of the medical students. In this context, the intervention might not have been precise, which could have affected the intervention efficacy. Moreover, except for MBSR, many of the interventions were not delivered by trained clinicians, which might lead to failure in emotion transmission that impairs teaching efficiency and the learning experience in medical education [24, 25]. Therefore, a problem-oriented educational intervention that precisely targets the specific medical student population with measurable short- and long-term efficacy should be explored and developed.

In China, medical students usually spend five years in their undergraduate courses and begin clinical work in their third year. In addition to the heavy academic burden, this school-clinic transition increases the study load and threatens mental well-being [17, 26]. Moreover, 3rd - and 4th -year students engage in direct professional patient-doctor communication. However, our university offers only some courses on the medical humanities for first-year students, and these courses mainly focus on theories of medical development and modern patient-doctor relations. Communication skills training is lacking for pre-clinical students. Higher frequencies of depression and burnout and lower empathy and sense of personal achievement have been reported in senior medical students compared to those in junior medical students [17, 18, 27]. Nevertheless, related randomized intervention studies carried out with Chinese medical students are very limited, and thus, they are urgently needed.

In the current study, we aimed to develop a set of physician-guided intervention courses and perform a prospective randomized controlled study to evaluate the efficacy of the intervention, to improve the mental well-being and empathy of 3rd - and 4th -year medical students in China.

## Methods

### Development of the intervention

To make the intervention courses context-specific, we first attempted to understand the psychological disturbances of the 3rd - and 4th -year medical students in our hospital. We randomly recruited five pre-clinical medical students (3rd year) and five interns (4th year) to participate in a pilot study for preliminary data collection to determine the themes of the intervention courses. We met with the pilot study participants and discussed their impressions of their current clinical work and related mental difficulties for three times within three months. The students often mentioned a lack of motivation and achievement in daily repetitive medical work, pressure to cope with the doctor-patient relationship and the need to acquire action-oriented strategies or skills to help them face the workload and potential medical negligence. Using an iterative process of coding scheme and discussion to resolve disagreements [28, 29], we summarized the key concepts and organized the topics into three modules: “Establishing a Sense of Achievement”, “Means for Efficient Patient-Doctor Communication”, and “Strategies to Manage Medical Errors”.

It has been reported that the narrative storytelling, a variant of appreciative inquiry, helped establish the students’ faith understanding and internalization [28]. Furthermore, in consideration of the importance of the context specificity and the emotion transmission, we therefore introduced three experienced clinicians to narrate stories that were related to the identified themes based on their own real clinical experience, including the positive aspects of medical practice, strategies for efficient patient communication and the responses to medical errors (Additional file 1).

Building on previous literature [30–32], we further included facilitated discussion groups that incorporated elements of mindfulness, shared experience, and small-group learning in the intervention. The same general structure was followed in each course: (1) check-in and welcome, (2) environment preparation (e.g., leisure time, creation of a friendly atmosphere, and a simple reflective exercise), (3) story narration, (4) skills and solutions learning, (5) facilitated group discussion, and (6) summary and checkout.

### Investigation of the intervention efficacy

#### Participants

This was a single-centre, randomized controlled study carried out in the Zhongshan School of Medicine, Sun Yat-sen University, between March 2016 and June 2016. The enrolment criteria included (1) third- and fourth-year medical students who (2) were studying major subjects of clinical medicine and (3) lacked a history of mental illness. Finally, a total of 146 medical students were enrolled. All the enrolled participants read and signed written informed consent before participating in the study.

#### Sample size

The power and sample size were calculated with a two-sided chi-square test with a 5 % significance level. We estimated that with 64 students assigned to each group, the study would have 80 % power to detect 5-point differences for the primary end point of empathy between the two groups, with a standard deviation of 10. In the current study, the sample size was 146, rendering 85 % power to detect a 2.5-point difference in the quality of life scale and 64 % power to detect a 15.7 % difference in depression between the two groups, with a standard deviation of 5.

#### Randomization

Eligible participants were randomly assigned to the intervention (*n* = 74) and control (*n* = 72) groups according to the randomization results, which were sealed in an opaque envelope delivered by the co-investigators. The randomization coding list was generated by a simple randomization allocation method, the PLAN procedure (SAS, version 9.4, USA). Each student was assigned a number, which was linked to the corresponding contact information kept by the third-party supervisor. Neither the students nor their teachers were blind to the allocated group due to the interactive schedules between the two groups.

#### Intervention and control

The intervention courses were delivered every month for three months after regular medical courses. Students in the intervention group were divided into three small groups (~ 25 students/group) based on the order of their code numbers, and each group was guided by a corresponding lecture. Each course lasted approximately 60 min. Students in the control group were also guided by a teacher and were encouraged to discuss their difficulties in clinical work and school life. This was done in a leisurely, friendly atmosphere. Correspondingly, the teacher shared related experiences or gave suggestions for dealing with these problems. However, there was no structural course form provided and no predefined topics were discussed.

#### Data collection

In this trial, we used five validated questionnaires to evaluate self-efficacy (SE), quality of life (QoL), depression, burnout and empathy. All participants were asked to complete the self-assessment scales and provide their individual randomized number on the questionnaires that were administered before and one month after the whole intervention courses. The following scales were used.

##### General self-efficacy scale

SE was measured by the General Self-Efficacy (GSE) scale. The GSE scale is designed to measure a broad, stable sense of personal competence to effectively deal with a variety of stressful situations [33]. The scale is composed of 10 itemized questions that are answered on a 6-point Likert-type scale ranging from strongly disagree (score 1) to strongly agree (score 6). A higher total score indicates a higher level of SE.

##### Medical outcomes study short form 8

QoL was measured by the Medical Outcomes Study Short Form 8 (SF-8). The SF-8 is a valid QoL survey that covers the same eight domains of the SF-36 but in a shorter (eight question) form [34]. The domains covered by the SF-8 Likert-type scale include physical function, limitations due to physical health problems, bodily pain, general health, energy/fatigue, social function, limitations due to emotional problems, psychological distress and mental well-being. The pain domain score ranges from 1 to 6, while the other domain scores range from 1 to 5. Higher scores represented better physical state and mental state.

##### Patient health questionnaire-9

Depression was measured by the Patient Health Questionnaire-9 (PHQ-9). The PHQ-9 has 9 questions assessing the following symptoms: depression (anhedonia and depressed mood), suicidal tendency (suicidal thoughts), physical symptoms (trouble sleeping or concentrating, fatigue, changes in appetite, and a feeling of being slowed down or restless) and feelings of guilt or worthlessness over the past two weeks. A total score of > 9 points or the presence of suicidal tendency indicated the existence of depressive symptoms.

##### Maslach burnout inventory

The Maslach Burnout Inventory (MBI) is a valid and widely used standard survey to measure burnout [35–37]. Burnout comprises three domains: emotional exhaustion (EE), depersonalization (DP), and personal accomplishment (PA). The MBI uses a 7-point Likert scale and includes 22 items evaluating all three domains (the score range for EE is 0–54, the score range for DP is 0–30 and the score range for PA is 0–48). Medical students who score ≥ 27 in the EE domain or ≥ 10 in the DP domain are identified as having at least one manifestation of professional burnout [38].

##### Jefferson scale of empathy-health care provider student version

We measured empathy with the Jefferson Scale of Empathy-Health Care Provider Student version (JSE-HPS), which includes 20 items answered on a 7-point Likert scale. The students were asked to score each item according to their level of agreement (from 1 = strongly disagree to 7 = strongly agree). Ten items are negatively worded and reverse scored. The total JSE-HPS score ranges from 20 to 140, with higher scores indicating a higher degree of empathy [39]. The itemized questions in each survey were initially translated into Chinese for data collection and then translated back into English by bilingual researchers at the First Affiliated Hospital of Sun Yat-sen University to ensure the accuracy of the translation. All of the questionnaires, including the General Self-Efficacy (GSE) scale [40, 41], the Medical Outcomes Study Short Form 8 (SF-8) [42], the Patient Health Questionnaire-9 (PHQ-9) [43–46], and the Jefferson Scale of Empathy-Health Care Provider Student version [27], have been validated for the Chinese population.

### Follow-up

To evaluate the long-term impact of the intervention courses, we contacted all the intervention group students two years after the intervention (June 2018) by e-mail. The students were requested to reflect on the intervention courses by responding to the following three items: (1) Please write down the most impressive story you remember from the intervention courses. (2) Which aspect of the intervention courses influenced you the most? (3) How did you apply the strategies from the intervention courses in your daily clinical practice? The third question was used to evaluate if the interventions were internalized in the students. We collected all the answers from the e-mail. The core sentences and words that indicated the student’s mental or emotional reflection on the intervention were extracted. These concepts were further discussed, classified and summarized by the authors.

### Statistical analysis

Questionnaires with missing information on three or more items were excluded from the subsequent analysis. If the scores of a few scale items were missing, the medians of the scores of the same items from the remaining students were used to replace the missing data. The baseline characteristics of the participants were described with the mean (SD) if the variable followed a normal distribution, and if the data did not follow a normal distribution, the median (IQR) was used. The independent sample t test or Wilcoxon rank sum test was used to compare differences in the continuous variables between two groups, while the chi-square test or Fisher’s exact test was used to analyse the categorical variables. The significant differences between the baseline and follow-up scores for each outcome for both the intervention and control groups were also determined using the paired t test, and the differences between the baseline and follow-up scores were calculated and compared. All of the statistical analyses were performed using SAS (version 9.4, USA). A *P* value < 0.05 was considered to be statistically significant.

## Results

### Cohort and baseline characteristics

**Fig. 1:**
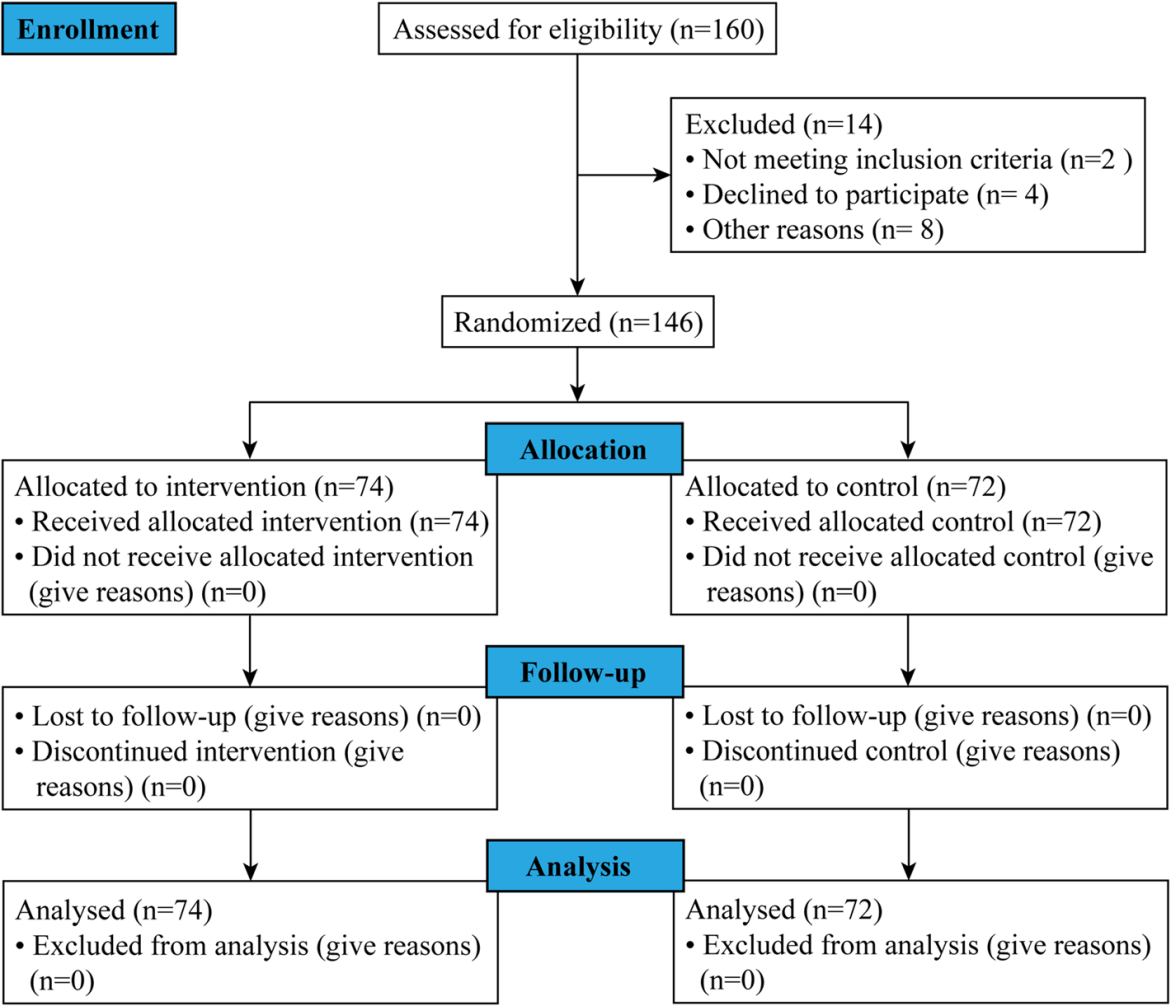
Flowchart of the inclusion of study participants according to CONSORT diagram

We initially recruited 160 3rd - and 4th -year medical students. After screening, a total of 146 students were enrolled and randomized to two arms (74 students in the intervention group and 72 students in the control group), as shown in Fig. 1. Among all collected surveys, 15 missing values were found, and none of the scales were excluded from the analysis. No significant differences in gender, grade or age were observed between the intervention and control groups. In addition, the baseline levels of SE (26.1 ± 4.4 vs. 25.8 ± 5.6; *P* = .76), QoL (31.0 [IQR 28.5, 34.0] vs. 31.0 [IQR 29.0, 34.0]; P = .62), depression (15.3 % vs. 24.3 %; chi-square test, *P* = .17), burnout (33.3 % vs. 35.1 %; chi-square test, *P* = .82) and empathy (110.5 [IQR 101.5, 119] vs. 110 [IQR 103, 116]; *P* = .98) between the two groups were not significantly different (Table 1).

**Table 1:** Baseline Characteristics of 146 senior medical students between intervention and control group

Variables	Total	Control group (*n* = 72)	Intervention group (*n* = 74)	*Statistics*	*P* value^a^
Age, year (range)	21.0 (20.0, 21.0)	20.5 (20.0, 21.0)	21.0 (20.0, 21.0)	3250	0.42
**Gender**				0.025	0.87
Males	76 (52.1 %)	37 (51.4 %)	39(52.7 %)		
Females	70 (47.9 %)	35 (48.6 %)	35(47.3 %)		
**Grade**					0.618
3rd	74 (51.4 %)	38 (52.8 %)	36 (48.6 %)		
4th	72 (48.6 %)	34 (47.2 %)	38 (51.4 %)		
SE, mean (SD)	26.0 (5.0)	26.1(4.4)	25.8(5.6)	0.360	0.76
QOL, median (IQR)	31.0 (29.0, 34.0)	31.0 (28.5, 34.0)	31.0 (29.0, 34.0)	2679	0.62
Depression (PHQ-9), mean (SD)	6.3 (3.4)	5.9 (3.0)	6.6 (3.7)	1.276	0.20
Depression (PHQ-9), N (%)				1.876	0.17
No	117 (80.1 %)	61(84.7 %)	56 (75.7 %)		
Yes	29 (19.9 %)	11(15.3 %)	18 (24.3 %)		
Burnout (MBI), mean (SD)	46.3 (14.7)	46.8 (14.6)	46 (15.0)	-0.336	0.74
Burnout (MBI), N (%)				0.053	0.82
No	96 (65.8 %)	48 (66.7 %)	48 (64.9 %)		
Yes	50 (34.2 %)	24 (33.3 %)	26 (35.1 %)		
Empathy (JSE-HPS), median (IQR)	110 (102, 118)	110.5 (101.5, 119)	110 (103, 116)	2514	0.98

Notes: Continuous variables are presented as mean (SD), median (IQR), and N (min, max). If the variable follows a normal distribution, it is described with the mean (SD) and if it does not follow a normal distribution, the median (IQR) is used. Categorical variables are presented as n (%) according to different levels

Abbreviations: *SD* standard deviation, *IQR* inter-quartile range, *SE* self-efficacy, *QOL* quality of life, *MBI* Maslach Burnout Inventory, *PHQ-9* the Patient Health Questionnaire-9, *JSE-HPS* the Jefferson Scale of Empathy-Health care Provider Student version

^a^t-test results for continuous variables with normal distribution, Wilcoxon test results for continuous variables with abnormal distribution, and Chi-square test results for categorical variables

### Short-term outcomes of the randomized arms

**Fig. 2:**
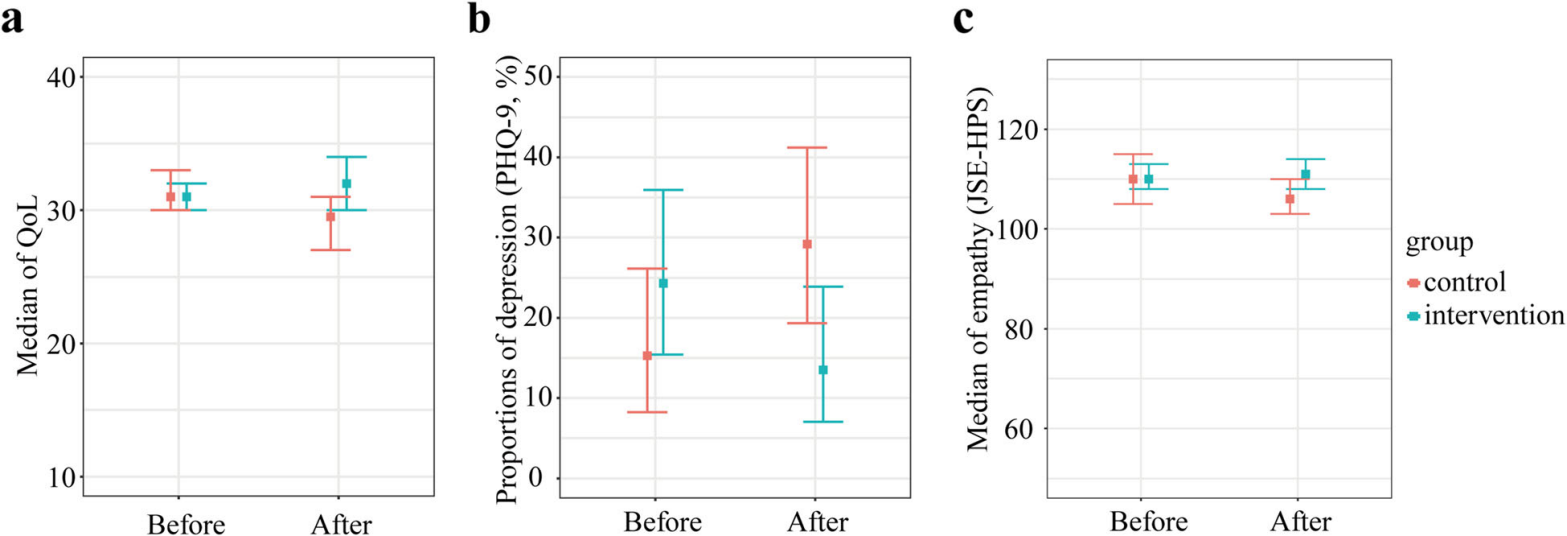
The scatter plot of (**a**) median of QoL, **b** proportion of depression (PHQ-9), and **c** median of empathy (JSE-HPS) before and after intervention for each group. Red represents data from control group and blue from intervention group. Bars mean 95 % confidence interval

Compared to the students in the control group, the students in the intervention group had significantly higher scores for QoL (32.0 [IQR 28.0, 35.0] vs. 29.5 [IQR 26.0, 34.0]; *P* = .04) and empathy (111.0 [IQR 102.0, 118.0] vs. 106.0 [IQR 93.0, 111.5]; *P* = .01) and a lower rate of depression (13.5 % vs. 29.2 %; chi-square test, *P* = .02) (Fig. 2; Table 2). The burnout score in the intervention group was lower than that in the control group (45.1 ± 13.6 vs. 49.6 ± 13.1; *P* = .04); however, no significant difference was observed in the burnout rate between the two groups (27.0 % vs. 34.7 %; chi-square test, *P* = .31) (Table 2). The students in the two groups had similar SE scores, with no significant difference (25.6 ± 4.8 vs. 24.3 ± 6.3; *P* = .16) (Table 2).

**Table 2:** Outcomes of 146 senior medical students between intervention and control group

Variables	Total	Control group(*n* = 72)	Intervention group (*n* = 74)	*Statistics*	*P* value ^a^
SE, mean (SD)	25.0 (5.6)	24.3 (6.3)	25.6 (4.8)	-1.40	0.16
QOL, median (IQR)	31.0 (26.0,35.0)	29.5 (26.0, 34.0)	32.0 (28.0, 35.0)	3089	0.04
Depression (PHQ-9), mean (SD)	6.7 (4.1)	7.6 (4.4)	6.0 (3.7)	-2.453	0.02
Depression (PHQ-9), N (%)				5.347	0.02
No	115 (78.8 %)	51 (70.8 %)	64 (86.5 %)		
Yes	31 (21.2 %)	21 (29.2 %)	10 (13.5 %)		
Burnout (MBI), mean (SD)	47.2 (13.6)	49.6 (13.1)	45.1 (13.6)	-2.087	0.04
Burnout (MBI), N (%)				1.014	0.31
No	101 (69.2 %)	47 (65.3 %)	54 (73.0 %)		
Yes	45 (30.8 %)	25 (34.7 %)	20 (27.0 %)		
Empathy (JSE-HPS), median (IQR)	108.5 (99.0, 115.0)	106.0 (93.0, 111.5)	111.0 (102.0, 118.0)	3195	0.01

Note: Continuous variables are presented as mean (standard deviation, SD) and median (inter-quartile range, IQR). If the variable follows a normal distribution, it is described with the mean (SD) and if it does not follow a normal distribution, the median (IQR) is used. Categorical variables are presented as n (%) according to different levels

Abbreviations: *SD* standard deviation, *IQR* inter-quartile range, *SE* self-efficacy, *QOL* quality of life, *MBI* Maslach Burnout Inventory, *PHQ-9* the Patient Health Questionnaire-9, *JSE-HPS* the Jefferson Scale of Empathy-Health care Provider Student version

^a^t-test results for continuous variables with normal distribution, Wilcoxon test results for continuous variables with abnormal distribution, and Chi-square test results for categorical variables

We also compared the baseline and follow-up scores for each outcome for both the intervention and control groups to determine whether there were significant differences. The data showed from baseline to one month after intervention, the control group had decreased scores for SE (1.79, [95 % CI: 0.01, 3.57], *p* = .05), QoL (1.9, [95 % CI: 0.23, 3.58], *p* = .027), and empathy (5.44, [95 % CI: 5.81, 10.08], *p* = .023); increased depression scores (-1.68, [95 % CI: -2.91, -0.4], *p* = .008); and unchanged levels of burnout (-0.54, [95 % CI: -4.46, 3.37], *p* = .787).

Compared to baseline, at one month after intervention, the intervention group had decreased depression scores (1.12, [95 % CI: 0.5, 2.45], *p* = .028) and unchanged levels of SE (0.15, [95 % CI: -1.5, 1.79], *p* = .862), QoL (0.48, [95 % CI: -0.97, 1.92], *p* = .52), empathy (2.04, [95 % CI: -1.69, 5.76], *p* = .023) and burnout (2.71, [95 % CI: -0.62, 6.03], *p* = .787).

### Long-term efficacy of the intervention courses

We obtained twenty-one replies at follow-up, for a response rate of 25.6 %. For the first item (Please write down the most impressive story you remember from the intervention course), it was striking that 15 of the 21 students referred to one of the stories from the “Means for Efficient Patient-Doctor Communication” module. The stories in the “Strategies to Manage Medical Errors” were recalled by five students, while one student was impressed with the story from the “Establishing a Sense of Achievement” module (Additional file 2). In response to the second question (Which aspect of the intervention courses influenced you the most?), all the students reported the positive aspects of the intervention courses and their positive and constructive influence on their daily practice and long-term careers. In addition to learning the strategies and skills, the students also reported the importance of learning how to establish an emotional connection with the patients. They reflected on the shift in their perception of doctor-patient relations and medical errors. In response to the third question (How did you apply the strategies from the intervention courses in your daily clinical practice?), most of the students reported that they learned to pay special attention to their teachers’ conversation skills, as well as the expressions and attitudes of patients and their families in their daily work. In addition, careful history taking, bodily examinations and multidisciplinary clinical decisions were also mentioned as important for reducing medical errors.

## Discussion

In this study, we investigated the impact of an educational intervention on mental health and empathy of senior Chinese medical students. Generally, the problem-oriented and physician-guided courses showed positive effects on students’ well-being, as evidenced by significantly improved QoL, depression and empathy.

In China, the 3rd and 4th years of clinical medical education are an important transitional stage. In our centre, 3rd - and 4th -year students have the heaviest academic load (e.g., they must pass the final examinations for 9 clinical subjects and various Objective Structured Clinical Examination [OSCE] tests). In addition, the 3rd - and 4th -year students are just at the very beginning of their clinical work and have little clinical experience in the communication with the patients, which can bring them a lot of stress. Interventions should be developed to help students go through this stage. Several previous trials have reported some useful methods that reduce distress or stress, such as MBSR-based exercises, psychological counselling and peer communication [19, 20, 22, 47]. Nevertheless, knowledge of mental problems or difficulties among this subset of Chinese medical students is limited. To make the intervention context-specific, we performed a pre-trial pilot study, which offered important clues indicating that these students need more clinical guidance and communication skill support, in addition to psychological comfort. The pilot study has provided direct data for the establishment of the precise intervention courses targeting this subset of students. The improved QoL, depression and empathy at one month after the intervention and the positive effects on the perception and clinical ability observed for some of the students after 2 years revealed the efficacy of the intervention courses. Our data provide evidence that the problem-oriented educational intervention might be useful. In addition to the reported methods, the method presented in the current study could be explored to address mental health or empathy problems in a specific medical student population.

Another difference in the design of the present study from that of most of the previous work is that all intervention courses are guided by experienced physicians. We take emotional transmission and sincerity into account in this trial since emotion has been found to have a positive effect on learning efficiency and internalization of clinical skills and knowledge [24, 48]. It is inspiring to find that the courses helped some of the students in their future clinical practice. This result indicates the importance of emotion and professionalism in interventions involving senior Chinese medical students.

Previous studies have shown that the prevalence of mental health problems in medical students was significantly higher than that in the general population and was even higher in senior medical students [49–51]. Similarly, we also observed a high prevalence of depression and burnout in this study. After the intervention, compared to the control group, the intervention group showed significant improvement in QoL and depression. Depression was also reduced after the courses compared to that at baseline in the intervention group, indicating a benefit in the relief of mental stress. Notably, we observed that the depression rate of students in the control group significantly increased. A possible explanation might be that the final exams were approaching, which placed a considerable amount of pressure on the students, as the 3rd- and 4th-year medical students in our university needed to pass 9 clinical subjects and various OSCE-based tests for their final examinations. Such pressure also likely explained why not only depression but also SE, QoL, and empathy worsened in the control group, while the mental health levels of the students in the intervention group showed improvement or unchanged levels after courses compared to those at baseline. This phenomenon might imply the positive effect of the intervention to help students face daily stress and maintain a stable mental state. In the study, SE and burnout did not show significant improvement, which could be attributed to the relatively short duration of the intervention courses. Future studies with longer intervention courses are required.

Empathy is another important ability of medical students, especially for senior Chinese medical students. Over the past 20 years, many violent incidents perpetrated by patients have been reported, which has made being a doctor in China a high-risk job [13, 26] and has affected students’ faith in their careers [15]. Poor doctor-patient relationships might lower students’ clinical empathy, which could further exacerbate doctor-patient communication, ultimately affecting patient prognosis [52, 53]. Empathy could be of great help in improving doctor-patient communication to achieve the best mutual understanding [54], and it has also been found to be important to patient care by enhancing patient satisfaction, comfort, self-efficacy and trust, leading to accurate diagnosis, shared decision making and therapy adherence [55]. In this study, we introduced patient-doctor communication skills by narrating and sharing real stories, eventually effectively improving the empathy of medical students in the intervention group compared to that of students in the control group. More importantly, as described in the follow-up e-mails, the students remembered the stories and emphasized skills learning two years after the courses had ended.

This study also has several limitations. First, this is a single-centre trial. Multicentre studies should be carried out in the future to further validate the efficacy of similar interventions. In addition, the Chinese versions of all five scales have been validated in previous studies [39–46]. However, it is difficult to guarantee the complete preservation of the original intention due to translation. Third, there are a few missing data for some scale items, but the proportion is less than 1 %. Another minor limitation is that it is difficult to prevent the potential communication between the students in the control and intervention group after courses. If the students in the intervention group had deep talk with the students in the control group, it might lead to bias of the short-term outcomes.

## Conclusions

Intervention courses guided by clinicians sharing real-life experiences significantly improve QoL, promote empathy and reduce depression among senior Chinese medical students. Our results pave the way for the development of a novel strategy for course reform in the education curriculum in Chinese medical schools by adding accessible, low­cost and easy-to-implement educational interventions to improve stress-related mental health and empathy.

## Supplementary Information

**Additional file 1.**

**Additional file 2.**

## Data Availability

The datasets analyzed during the current study are available from the corresponding author on reasonable request.

## Abbreviations

GSE: General Self-Efficacy
SF-8: Medical Outcomes Study Short Form 8
PHQ-9: Patient Health Questionnaire-9
MBI: Maslach Burnout Inventory
JSE-HPS: Jefferson Scale of Empathy-Health care Provider Student version
SE: Self-efficacy
QoL: Quality of life
MBSR: Mindfulness-based stress reduction
EE: Emotional exhaustion
DP: Depersonalization
PA: Personal accomplishment
SD: Standard deviation
IQR: Inter-quartile range
OSCE: Objective Structured Clinical Examination
