# Supplementary material for: Improvement of the management of mental well-being and empathy in Chinese medical students: a randomized controlled study
Source: BMC Med Educ. 2021 Jul 10;21:378. doi: 10.1186/s12909-021-02813-6 (PMC8272356; doi:10.1186/s12909-021-02813-6)
Supplement: Supplementary file 1 — Additional file 1. [file 12909_2021_2813_MOESM1_ESM.docx]

**The contents of the intervention courses**

The intervention courses focused on three main subjects: (1) Establishing a Sense of Achievement, (2) Means for Efficient Patient-Doctor Communication, (3) Strategies to Manage Medical Errors.

In the “Establishing a Sense of Achievement” module, we hoped to communicate four positive aspects of medical practice to our students, including the professional ability of physicians to save lives, the meaning of gratitude or good faith from the patient, the importance of newly acquired clinical knowledge from the daily work and one’s reputation earned among colleagues. These stories were intended to help the students establish good feedback regarding cognition and behaviour in their daily clinical study and work.

In the “Means for Efficient Patient-Doctor Communication” module, we emphasized the importance of the art of patient-doctor conversation and the ability to show empathy. The lecturer shared some successful examples. These examples always involve some conflicts, for instance, between the expensive treatment cost and poor economics of the patient’s family, etc. In these examples, the physicians gave appropriate and effective communication with the patients or their families. As a result, the patients or their family members could fully understand the therapeutic decision and did not complaint the therapeutic outcomes. The communication skills and empathy reflected in these vivid stories were intended to help our students optimize clinical decisions and advice based on respect for patients.

In the “Strategies to Manage Medical Errors” module, the lecturer shared medical errors that she had made and the strategies she used to prevent and solve similar problems in her future work. These stories were important to tell our students to communicate that everyone can make mistakes, even a very experienced doctor. In addition, it was essential for the students to learn skills for reporting mistakes to a superior doctor and communicating with patients and their family members after medical errors. These stories were intended to help the students establish proper attitude and confidence towards the medical errors.

Three experienced clinicians with at least 10 years of clinical service in our affiliated hospital were selected to serve as lecturers. The lecturers were trained by a member of our team to ensure that they were familiar with the protocols and were able to narrate the stories fluently.
