# Supplementary material for: Improvement of the management of mental well-being and empathy in Chinese medical students: a randomized controlled study
Source: BMC Med Educ. 2021 Jul 10;21:378. doi: 10.1186/s12909-021-02813-6 (PMC8272356; doi:10.1186/s12909-021-02813-6)
Supplement: Supplementary file 2 — Additional file 2. [file 12909_2021_2813_MOESM2_ESM.docx]

**A brief quotation from the story that most impressed the students.**

“I met one patient who was suffering from chronic liver disease, and his condition was turning relatively serious. The necessary treatment was very expensive. His wife asked me whether she should continue the treatment because they had three kids to raise and the current family economic situation was poor. The first step I took was to tell her all the advantages and disadvantages of the continuing treatment within the scope of my professional requirement. Next, I gave her my own opinion: ‘If I were you, I would continue the treatment as long as the charge wouldn’t bankrupt us and hurt our family’. In the end, I fully understood the woman’s tough situation, so I gently told her, ‘I know your pain, so I would understand and respect your final decision whatever you selected’. Please never try to morally comment or to condemn the patient’s or the family members’ choice but learn to think and communicate from the perspective of patients. In the subsequent treatment, both the patient and his wife trusted me a lot.”

**Some representative quotes from the participants’ replies.**

“I still remember one story: when the patient’s wife asked whether she should keep trying to treat her chronically ill husband with a very large cost, and the teacher answered, ‘If I were you, I would try to save him as long as there is hope and comfort for him without going bankrupt and hurting yourself.’ I found this story very touching, and I am now using this way of discussion in my clinical practice when facing a similar situation.”

“I remembered sharing my fears of making clinical mistakes, and my course teacher told us not to be afraid of making mistakes because a clinical decision is always made in the consensus view of the clinical team. We are always supported by our senior colleagues when facing new and complex clinical situations. The courses have provided me courage, clinical empathy and confidence to learn new techniques and explore novel surgical operations for the best prognosis of patients’ care.”
